# Validating Numerical Simulations to Support Experimental Testing of MRI Gradient‐Induced Heating of Passive Implants

**DOI:** 10.1002/mrm.70306

**Published:** 2026-02-15

**Authors:** Umberto Zanovello, Alessandro Arduino, Luca Zilberti, Gregor Schaefers, Martin Halaj, Oriano Bottauscio

**Affiliations:** ^1^ Istituto Nazionale di Ricerca Metrologica (INRiM) Torino Italy; ^2^ MR:comp Gelsenkirchen Germany; ^3^ Slovak University of Technology (STU) Bratislava Slovakia

**Keywords:** heating, implant labeling, magnetic resonance imaging, passive implants, switching gradient field, validation, virtual experiments

## Abstract

**Purpose:**

Numerical simulations can be adopted to aid the evaluation of the interaction between switched gradient fields and metallic implants and estimate the possible temperature increase. Anyway, an analysis of the consistency of their prediction with experiments is lacking, differently from what can be found for exposure to radiofrequency fields. The purpose of this work is to fill this gap.

**Methods:**

A systematic comparison between experiments and simulations was conducted, considering commercial metallic orthopedic implants. The complexity of the exposure scenario was gradually increased, starting from the conditions defined in the ISO/TS 10974:2018 standard and moving to more realistic exposure scenarios with sinusoidal or pulsed gradient fields.

**Results:**

The computational tools demonstrated an overall good capability in predicting the outcomes of the experimental tests, as long as numerical simulations are properly set up (building of the virtual model, discretization parameters, positioning/orientation of the object). The consistency between simulations and measurements is comparable with the one obtainable under radiofrequency exposure and decreases with exposure complexity.

**Conclusion:**

The results pave the way for the use of numerical simulations to support laboratory testing of passive implants heating under time‐varying gradient fields.

## 1 Introduction

Although magnetic resonance imaging (MRI) is generally safe, significant efforts are made to ensure the safety of patients with implanted medical devices. Metallic implants can interact with radiofrequency (RF) and switching gradient magnetic fields [1].

The RF field is scattered by the presence of the metallic components, resulting in power deposition which may concentrate near the implant extremities. RF is the most important source of heating in an MRI scanner.

Switching gradient fields have complex time waveforms with fundamental frequencies in the kilohertz range and harmonics extending up to tens of kilohertz. Eddy currents are induced in the metallic implant, generating heat that diffuses towards the surrounding tissues. A recent comparison [2] showed that the heating induced by switched gradient fields in bulky conductive objects can be comparable to the heating originating from the RF field.

Testing procedures have been defined in the standards to evaluate the implant heating. The ASTM F2182‐19 guidelines only cover the testing of passive implants under RF exposure [3], whereas the current ISO/TS 10974 standard [4] only covers gradient‐induced heating for active implantable medical devices exposed to the field of a 1.5 T whole body scanner. A new version of the latter has been drafted and will extend the current standard to 3 T scanners. A recent EU project [5] has sought to contribute to the expansion of standard documents to include the testing of passive implant heating when exposed to switched gradient fields.

Simulations can support experimental measurements and correlate heating results with those expected in the human body. They can be used to optimize experiments by determining the optimal measurement points, reducing the testing burden, and helping to define the worst exposure conditions. This is particularly useful when dealing with implants of complex geometries [6]. Simulations can also be used for analyzing the factors influencing heating and for studying the impact of individual parameters such as material properties, object shape and size, and tissue properties.

Validating numerical simulations is an essential step for their usage as surrogates of measurements [7]. This process aims to estimate predictive accuracy of the model when used to analyze the specific conditions of application.

Several studies have addressed the evaluation of simulation reliability with respect to RF field exposure [8, 9]. Conversely, experimental validation of switched gradient field heating was conducted only by using simple objects [10, 11] and realistic implant component heating was just demonstrated for a *proof‐of‐concept* case [12, 13].

Following [14], this work compares simulation and measurement outcomes to check if modeling approaches can support switched gradient field heating experiments. The analysis considers commercial orthopedic implants with exposure conditions of increasing complexity.

First, numerical simulations replicate the exposure conditions specified in the ISO standard, whereby the implants are subjected to a spatially uniform sinusoidal magnetic field aligned in a direction that maximizes heating.

In a second step, a comparison is performed in more complex exposure conditions. First, the implants are exposed to a spatial gradient field with a sinusoidal time waveform. Then, the exposure is performed with a waveform mimicking an MRI sequence which proved to be more aggressive in terms of heating.

## 2 Methods

### 2.1 Orthopedic Implants

**FIGURE 1:**
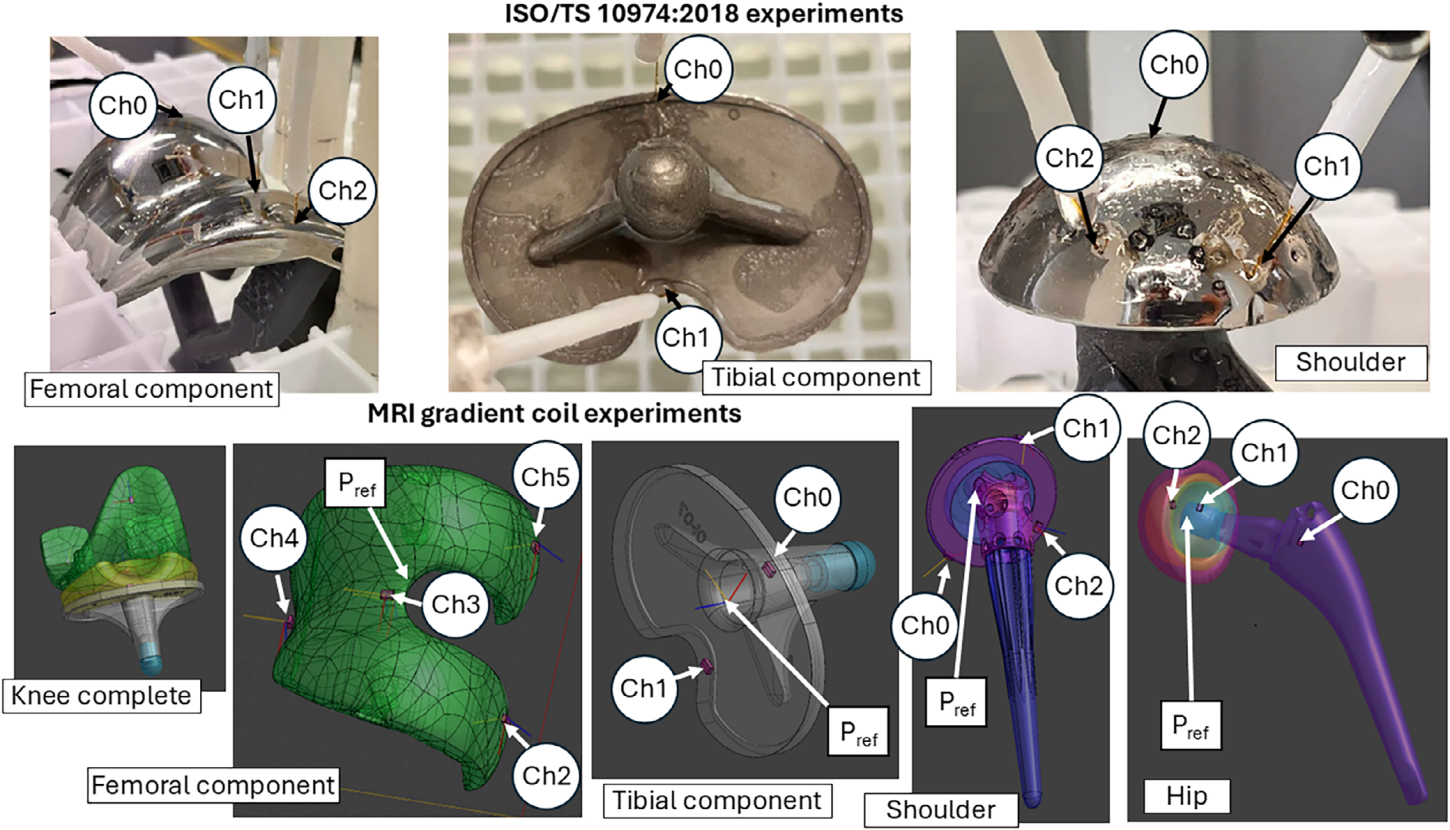
The top row shows photographs of the implants involved in the ISO 10974 experiments with the position of the thermal sensors indicated with labels. The bottom row shows the CAD models of the implants used in the experiments inside the MRI gradient coils. Also here the position of the thermal sensors is indicated with labels. The point P_ref_ is used as a reference to locate each model in a given position within the gradient coil system. Note that the thermal sensor labeling in the ISO/TS 10974:2018 experiments is different from the one used in the MRI gradient coil experiments.

Three implants were analyzed: knee, shoulder, and hip implants. CAD files of shoulder and hip implants were provided alongside the physical specimens, whereas the knee implant's CAD file was obtained through 3D scanning. Details are summarized in the Data S1 (Supporting Information). Figure 1 shows the implants used in the two experimental set‐ups.

In all the experiments, the device under test (DUT) was immersed in an acrylic phantom filled with a gel, prepared according to the recipe suggested by ISO/TS 10974:2018 [4] (see Data S2). Some experiments were also conducted independently on the two main bulk components (tibial plate and femoral) for the knee implant.

### 2.2 Computational Tools

Two computational tools were adopted for numerical simulations to highlight the impact of the geometrical discretization with complex‐shaped implants.

The first approach used a voxelized discretization (voxel‐based model), typically applied for in silico simulations involving digital anatomical models. The simulation tool consists of a first module, based on hybrid finite element–boundary element method, for solving eddy‐current problems in the frequency domain including skin effect in highly conductive materials [15] and handling non‐sinusoidal waveforms (e.g., pulsed gradient fields) [16]. A second module for solving the thermal problem is based on the finite difference method and the Douglas–Gunn time‐splitting scheme [17]. Both modules were implemented in Matlab 2022a (The MathWorks Inc.) environment, with submodules written in the Mex‐Fortran and CUDA‐C languages to efficiently handle GPU computations. The voxel size can differ between the two solvers.

The second approach used the Simulia CST Studio Suite 2025 [18], with non‐structured tetrahedral (TET) discretization (TET‐based model). The uni‐directional coupled tool combined the low frequency domain solver, which accounts for skin and proximity effects, and the thermal transient solver.

### 2.3 Experimental Facilities

#### 2.3.1 Experiments Following ISO/TS 10974:2018 Test Method

These experiments were conducted at the MR: comp laboratories using a Pulsed Magnetic Field Simulator and applying the ISO/TS 10974:2018 test method. Tests were conducted on the shoulder implant and on the femoral and the tibial components of the knee implant.

A cylindrical phantom container (diameter: 190 mm; height: 245 mm) was used and the DUT was mounted on a polylactic acid (PLA) holder, which did not affect the gradient field exposure.

The implant was exposed to a linearly polarized, harmonic, homogeneous magnetic field, oriented in a direction that maximized heating (determined according to the algorithm described in [19]).

Two series‐connected coils generate a sinusoidal field oriented primarily along the coil axis at a frequency of 1750 Hz and an amplitude of 5.6 mT, achieving the *dB*/*dt*_RMS_ of 42 T/s required by the standard. Each test object was exposed to an 1800 s continuous sinusoidal sequence.

A fiber optic temperature system (Neoptix Inc.) was used to measure the temperature at the locations of the hotspots previously determined by simulations. The probes were accurate to ±0.1°C at the calibration point of 23°C.

Before the experiments, the saline gel was homogenized by stirring and its thermalization to room temperature (±1.0°C) was monitored using a mercury lab thermometer (Amarell, Germany). Room temperature was monitored prior to and after each test run to maintain constant environmental conditions.

The temperature on the implant surface was recorded for 2 min before the test, during the test and for another 2 min after the test, in recording intervals of 1 s. See Data S2 for details about measurement uncertainty.

#### 2.3.2 Experiments Using MRI Gradient Coils

Experiments were conducted at the INRiM laboratories using actively shielded whole‐body three‐axial gradient coils. Coils were supplied by a high‐power NG500 1.3 gradient amplifier cabinet (Prodrive Technologies) to generate gradient magnetic fields along three Cartesian directions. The spatial magnetic field was preliminarily characterized using a NARDA HP‐01 magnetometer.

Each DUT was placed in a cuboid phantom container with size of 130 mm × 440 mm × 200 mm. The DUT position was chosen to expose it to the maximum available field amplitude within the coils compatible with a realistic anatomical position during an MRI scan.

A fiber optic temperature system (Opsens TMS‐8) was used to measure the temperature of DUT hotspot locations. The probes were accurate to ±0.15°C at the calibration point of 23°C. Temperature was recorded for 30 s before and during the test in 2‐s intervals. An additional probe was placed in the gel close to the phantom wall to monitor that its temperature did not increase during the experiment. Details are reported in Data S3 and related measurement uncertainty is described in the Data S4.

In the first set of experiments, axes *X* and *Z* were simultaneously supplied with a sinusoidal current at 1750 Hz for 900 s. Tests were conducted on all three entire implants, plus two cases with the femoral and the tibial components of the knee implant, separately. The *dB*/*dt*_RMS_ values, evaluated in reference points P_ref_ of Figure 1, for each investigated implant position were: 30.8 T/s for the shoulder, 23.6 T/s for the knee, 23.5 T/s for the femur component, 23.9 T/s for the tibial component, and 24.8 T/s for the hip.

In a second set, a trapezoidal waveform was adopted, similar to the frequency encoding signal of an echo planar imaging sequence for 400 s, supplying the gradient coil which maximized the power deposition. This sequence is known to result in high power deposition [13]. The *dB*/*dt*_RMS_ values corresponding to the investigated implant positions were respectively: 26.2 T/s for the shoulder, 18.9 T/s for the knee, and 20.0 T/s for the hip.

### 2.4 Validation and Uncertainty Quantification (VUQ)

In general [14] identifies three sources of predictive error: (a) *numerical errors*, introduced by numerical solving algorithms, (b) *epistemic errors*, due to incomplete, idealized, or partially fallacious knowledge of the phenomenon, (c) *aleatoric errors*, due to the propagation of the measurement errors in model inputs.

In this analysis, convergence of modeling results was preliminary checked reducing the numerical errors to be negligible. For the voxel‐based model, the voxel size was set to 0.9 mm in the thermal solver and 0.3 mm in the electromagnetic solver to properly capture the eddy current path. The TET‐based model used an adaptive discretization scheme adjusting the mesh size to achieve a final solver relative accuracy of 10^−6^.

The propagation of input data measurement uncertainty is out of the scope of the present analysis, assuming input quantities are perfectly known. The attention is entirely focused on validation, to quantify the *epistemic errors*.

The highest level of credibility testing of the model is expected when it can accurately predict the entire population of measurements. A global predictive inaccuracy ***ε*** is estimated as the 2‐norm of the vector of differences between the predicted value, *p*_i_, and the measured value, *m*_i_ (with its uncertainty *u*(*m*_i_)) for each member of the population. Being affected by measurement uncertainty, ***ε*** is represented by a statistical distribution with associated 95% coverage interval.

## 3 Results

### 3.1 Exposure Under ISO/TS 10974:2018 Testing Conditions

At the end of the experiment (1800 s), the maximum simulated temperature increase was found to be 8.0 and 7.1 K for the femoral component, 5.3 and 5.7 K, for the tibial plate, and 4.5 and 4.1 K, for the shoulder implant, using the voxel‐based and TET‐based models, respectively. The ratio of the maximum temperature values of the voxel‐based model versus the TET‐based model ranged from 0.93 to 1.12 (see Table S3). Corresponding maximum measured temperature increases were (7.3 ± 1.1) K, (5.9 ± 0.9) K and (4.0 ± 0.6) K. The maximum heating is approximately located in the same spots in both modeling tools.

The total power dissipated in the implant components was 3.13 W (resp. 2.89 W) for the femur, 1.67 W (resp. 1.75 W) for the tibial plate, and 1.14 W (resp. 1.12 W) for the shoulder according to the voxel‐based (resp. TET‐based) model. The ratio between the total power computed by the two modeling approaches ranged from 0.96 to 1.08. See the Data S5 for more details.

**FIGURE 2:**
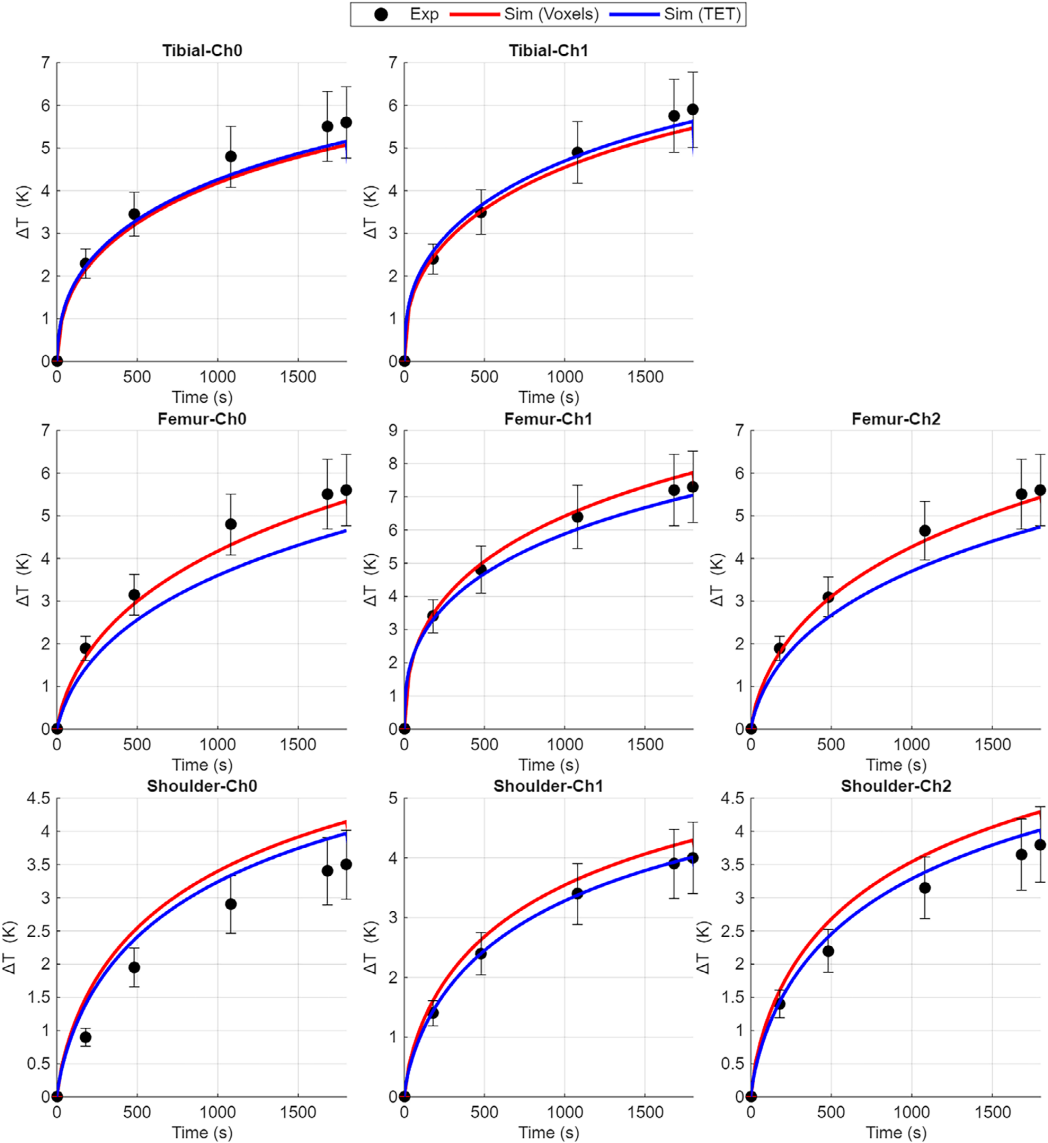
Measured and computed time evolution of the temperature increase for all DUTs. For each DUT and sensor, the curves obtained by the simulations (with voxel‐based and TET‐based models) are reported as solid lines. A selection of measured instant values, extracted from the experimental records, are plotted together with the corresponding expanded uncertainty bars (95% level of confidence).

Figure 2 shows the time evolution of the temperature increase computed by the voxel‐based and TET‐based models at the selected measurement points of the femoral component, tibial plate, and shoulder. In the same figure, the measurement results are reported for comparison.

Having verified the agreement between voxel‐based and TET‐based results, the successive analysis was performed using only the former method, more commonly adopted in MRI dosimetry.

### 3.2 Exposure to MRI Gradient Coils (Sinusoidal Time‐Waveform)

The energized *X* and *Z* axes generated field gradients of 8.2 and 8.5 mT/m, respectively, with magnetic field components depending on the position of the implant (see Data S6).

**FIGURE 3:**
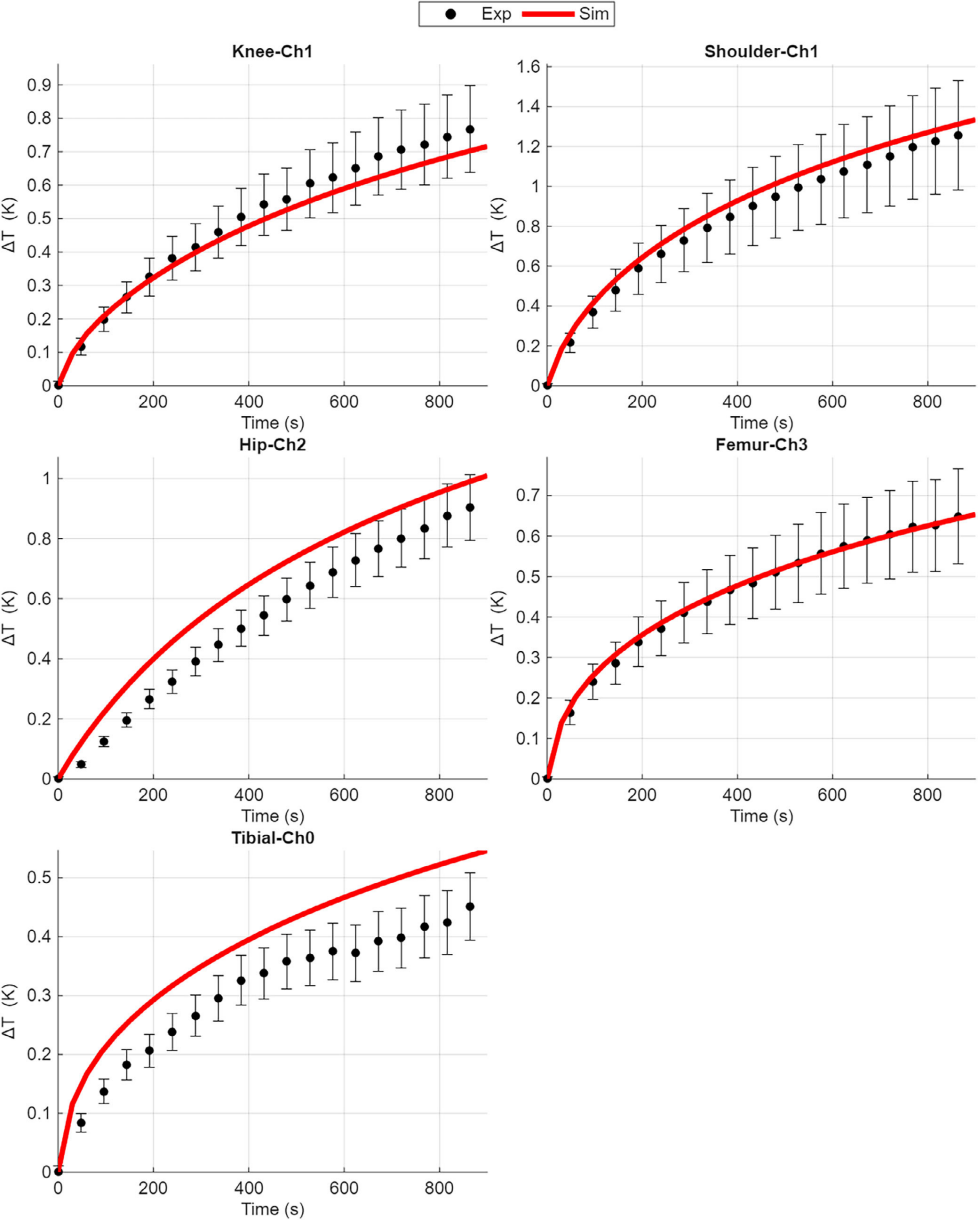
Time evolution of the temperature increase for all DUTs under exposure to sinusoidal time‐waveform non‐uniform magnetic field. Experiments with only tibial and femoral components are also reported. For each DUT the sensor which exhibits the maximum temperature increase is reported. The curves obtained by the voxel‐based simulations are reported as solid lines. A selection of measured instant values, extracted from the experimental records, are plotted together with the corresponding expanded uncertainty bars (95% level of confidence).

Figure 3 shows the time evolution of the temperature increase computed by the voxel‐based model and the measurement results at the measurement points which exhibit the maximum temperature increase for each DUT. The time evolutions for all sensors are reported in the Figure S5.

The total power dissipated in the implant components estimated by the voxel‐based model was 0.59 W for the entire knee, 0.36 W for the femoral component, 0.25 W for the tibial component, 0.46 W for the shoulder, and 0.58 W for the hip implant.

### 3.3 Exposure to MRI Gradient Coils (Trapezoidal Time‐Waveform)

The same implant positions selected for the sinusoidal field exposure were considered.

The trapezoidal time waveform of the gradient field had a fundamental frequency of 961 Hz and a frequency spectrum up to 14.4 kHz. The field gradient amplitude was 28 mT/m. See the Data S7.

**FIGURE 4:**
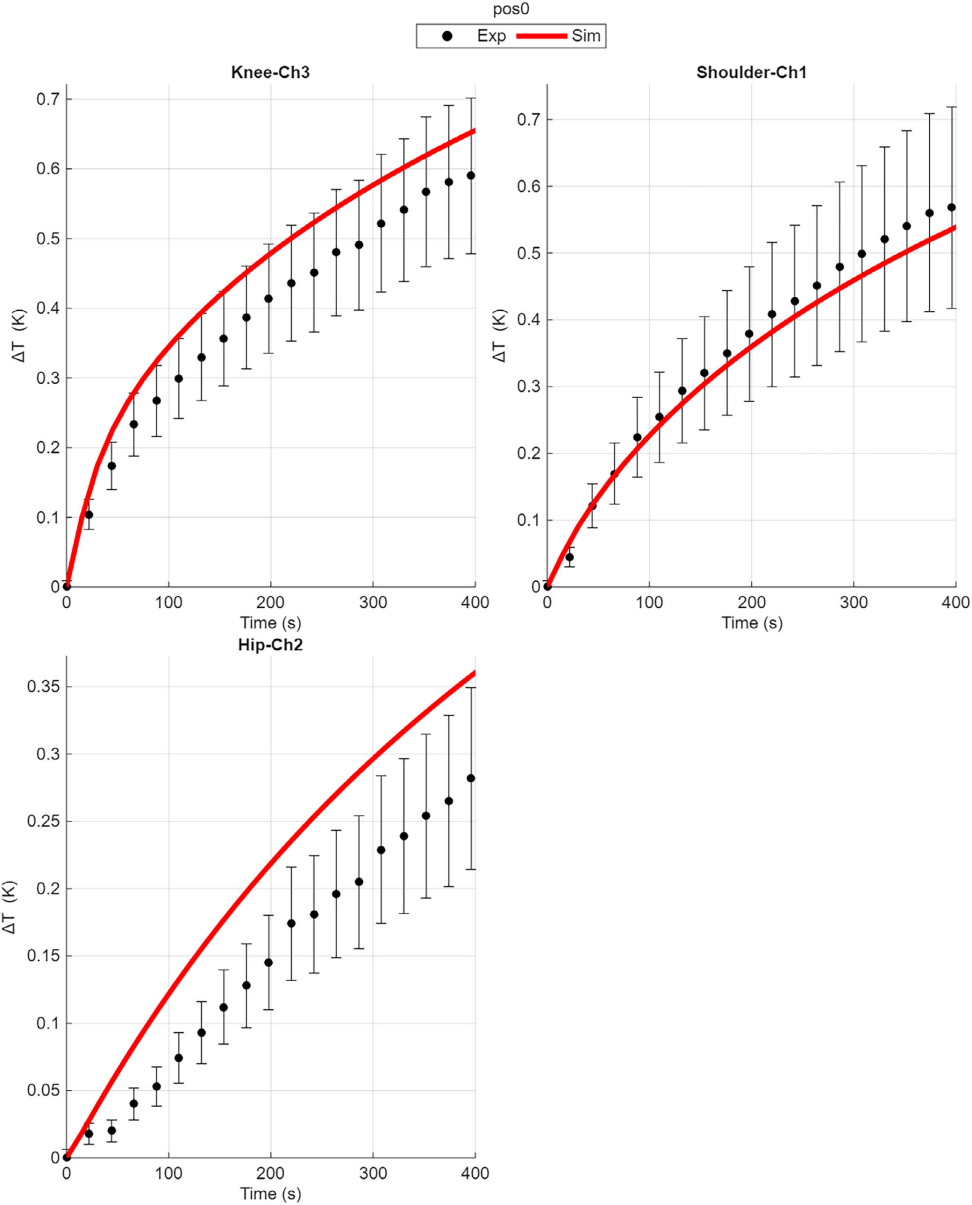
Time evolution of the temperature increase for all DUTs under exposure to trapezoidal time‐waveform non‐uniform magnetic field. The same DUT positions of the exposure with sinusoidal waveform are reported here. For each DUT the sensor which exhibits the maximum temperature increase is reported. The curves obtained by the voxel‐based simulations are reported as solid lines. A selection of measured instant values, extracted from the experimental records, are plotted together with the corresponding expanded uncertainty bars (95% level of confidence).

Figure 4 shows the time evolution of the temperature increase computed by the voxel‐based model and the measurement results at the measurement points which exhibit the maximum temperature increase for each DUT. The time evolutions for all sensors are reported in the Figure S7.

The total power dissipated values estimated by the voxel‐based model were 0.64 W for the knee implant, 0.30 W for the shoulder implant, and 0.36 W for the hip implant.

### 3.4 Dataset for the VUQ Process

The model accuracy is evaluated by comparing the simulated and experimental temperature increases in two ways: (a) at the end of the experiment (1800 s for the ISO/TS 10974:2018 test, and 900 s or 400 s for the GC heating with sinusoidal or trapezoidal waveform, respectively), as relevant during experimental testing, (b) for all sampled time steps (to account for possible inaccuracies during the transient evolution).

**FIGURE 5:**
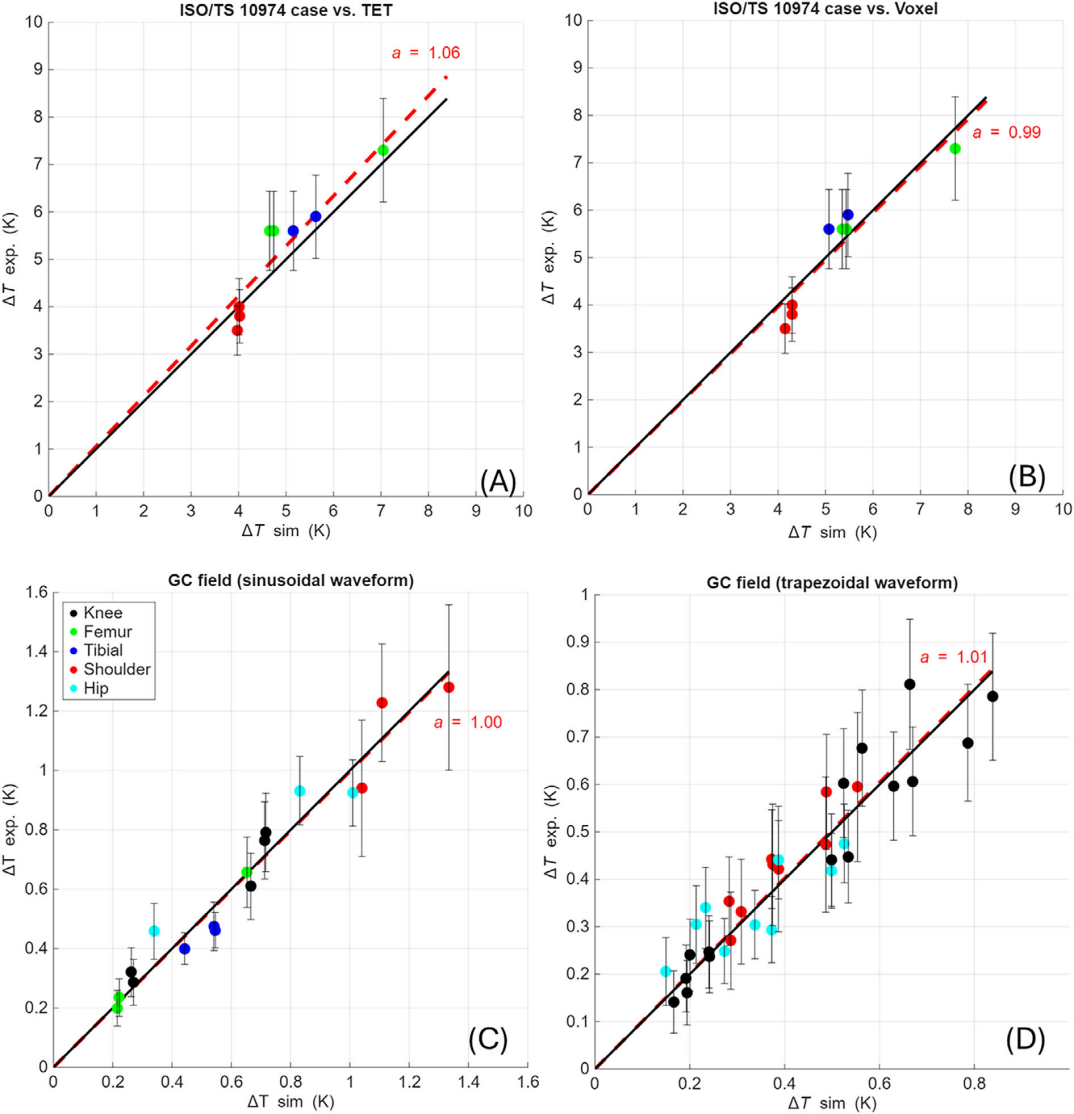
Comparison between simulated and measured values of the temperature increase at the end of the experiment for all the considered exposure conditions: (A, B) experiments following ISO/TS 10974:2018 test method, extracted from results reported in Figure 2 (A: comparison with TET‐based simulations; B: comparison with voxel‐based simulations), (C) experiments with gradient coils and sinusoidal waveform, extracted from results reported in Figure S5, (D) experiments with gradient coils and trapezoidal waveform, extracted from results reported in Figure S7. In each plot, on the *x*‐axis the simulated values (∆*T* sim), on the *y*‐axis the measured values (∆*T* exp). For each experimental value, the expanded uncertainty bar (95% level of confidence) is reported. The solid black line shows the bisector, which represents the ideal correspondence between measured and computed values. The red dashed line is the regression curve (slope *a*) computed assuming a linear model with null intercept.

Figure 5 shows the measured temperature increases at the end of exposure against the corresponding computed values for each scenario. A linear regression, weighted with measurement uncertainty, is computed assuming a linear model with zero intercept (slope equal to *a*). Most of the measurement points (with their uncertainty bars) intersect the regression line. Very few cases without intersection can be considered outliers.

## 4 Discussion and Conclusion

For ISO/TS 10974:2018 experiments, the values of parameter *a* are 1.06 and 0.99 for the TET‐ and voxel‐based models, respectively, quite close to the bisector line (*a* = 1). The corresponding global predictive inaccuracies ***ε*** (related to the temperature at the end of the experiments), expressed in percentage, have the statistical distribution reported in Data S8. The mean values and 95% coverage intervals are 10.8% and [6.28% ÷ 15.2%] for the voxel‐based model and 12.1% and [7.67% ÷ 16.5%] for the TET‐based model.

The predictive inaccuracies ***ε*** (mean values and coverage intervals) considering all sampled time instants are: 11.4% [9.26% ÷ 13.6%] for the voxel‐based model and 13.2% [10.9% ÷ 15.4%] for the TET‐based model.

These results demonstrate good overall agreement between simulations and experiments, with slightly better performance of the voxel‐based model compared to the TET‐based model.

For the exposure with gradient magnetic field, the regression parameter remains identical to the bisector line (*a* = 1.0) for the sinusoidal waveform. The predictive inaccuracy ***ε*** results in a mean value of 13.3% and coverage interval of [8.50% ÷ 18.1%], for the values at the end of exposure, and 15.4% [14.4% ÷ 16.5%] when all time instants are considered.

When considering trapezoidal time waveforms, the agreement between simulations and experiments further decreases. The regression parameter does not change significantly (*a* = 1.01), but the predictive inaccuracy ***ε*** increases up to 18.1% [14.4% ÷ 21.7%] when considering values at the end of experiment and 20.6% [19.8% ÷ 21.4%] when all time instants are considered.

As expected, inaccuracy therefore increases when transitioning from the simpler ISO/TS 10974:2018 exposure scenario to more realistic imaging conditions.

When evaluating the consistency between simulations and experiments, it should be noted that both are assumed to represent the best obtainable state‐of‐art performance. In performing the comparison, a preliminary check is required to assess the stability of numerical solutions in terms of spatial/time discretization and solver convergence. Indeed, the spatial resolution of electromagnetic and thermal solvers (∆s_EM_ and ∆s_TH_, respectively) impacts the simulation accuracy. The effect on the global predictive inaccuracy ***ε*** (with data at the end of experiment) was verified by repeating the simulations with a sinusoidal gradient magnetic field. When ∆s_EM_ was changed from 0.3 to 0.9 mm (while keeping ∆s_TH_ at 0.9 mm), the regression parameter remained close to 1 and a limited impact was found on ***ε*** (mean value: 13.7% and coverage interval: [9.13% ÷ 18.3%]). Similar results were obtained changing ∆s_TH_ from 0.9 to 3 mm while keeping ∆s_EM_ at 0.3 mm; the regression parameter remained close to 1 and a limited impact was found on ***ε*** (mean value: 13.5% and coverage interval: [9.60% ÷ 17.4%]). A larger increase of the predictive inaccuracy ***ε*** was found using both the higher spatial resolutions, ∆s_EM_ = 1.2 mm and ∆s_TH_ = 3 mm, with ***ε*** having a mean value of 17.6% and coverage interval of [14.1% ÷ 21.2%].

One of the most critical issues in numerical simulations is reproducing the actual experimental position and orientation of the DUT. The position implies different magnetic field amplitudes. Even more critical is the orientation of the object, which affects simulation results in a homogeneous magnetic field. Reproducing the actual object orientation is of paramount importance, especially when dealing with devices of complex shapes (e.g., knee implants) or components with multiple degrees of freedom (e.g., the hip joint). Further investigation is required to quantify the impact of these input data uncertainties.

Another important aspect is the correlation between the temperature measurement point in the experiments and the predicted location in the simulations. In all experiments, the active part of the sensor tip did not have perfect contact with the metallic surface of the implant. Instead, it measured an average temperature in a small volume (a 1 mm × 2 mm × 3 mm rectangular box) of the gel. This effect was considered in the uncertainty budget for the measured value by estimating the spatial distribution of temperature near the measurement point through numerical simulations.

The results suggest that numerical simulations can be reliably adopted to support measurements under highly controlled testing conditions, such as those prescribed in the ISO/TS 10974:2018 testing scenario. The predictive inaccuracy is lower than 20%, which is comparable to the measurement uncertainty. After validating the modeling tools for the specific use case, possible applications include determining the worst orientation of the DUT and evaluating hotspots to locate temperature sensors.

Finally, it should be noted that the focus of this work was on reproducing implant heating testing rather than predicting heating in human tissue. For this reason, the effects of the interface between the implant surface and human tissues were not considered, and only the interface between the implant and gel was studied, as well as the role of implant coatings or rough surfaces.

## Funding

This work was supported by European Partnership on Metrology, 21NRM05 STASIS.

## Conflicts of Interest

Gregor Schaefers has a relationship with MR:comp GmbH and MRI‐STaR GmbH that is a leading company for MR safety and MR compatibility testing of implants, instruments and medical devices.

## Supporting information


**Data S1:** mrm70306‐sup‐0001‐Supinfo.docx.

## Data Availability

The data that support the findings of this study are available at https://doi.org/10.5281/zenodo.18493595

## References

[1] L. Winter , F. Seifert , L. Zilberti , M. Murbach , and B. Ittermann , “MRI‐Related Heating of Implants and Devices: A Review,” Journal of Magnetic Resonance Imaging 53 (2021): 1646–1665, 10.1002/jmri.27194. 32458559

[2] U. Zanovello , A. Arduino , C. Fuss , T. Goren , L. Zilberti , and O. Bottauscio , “Impact of Simultaneous Exposure to RF and Gradient Electromagnetic Fields on Implant MR Safety Labeling,” Magnetic Resonance in Medicine 95 (2026): 601–612, 10.1002/mrm.70059. 40883877 PMC12620153

[3] American Society for Testing and Materials (ASTM) , Standard Test Method for Measurement of Radio Frequency Induced Heating on or near Passive Implants during Magnetic Resonance Imaging. Standard F2182‐19 (American Society for Testing and Materials (ASTM), 2019).

[4] International Organization for Standardization (ISO) , Assessment of the Safety of Magnetic Resonance Imaging for Patients With an Active Implantable Medical Device. Standard ISO/TS 10974 (International Organization for Standardization (ISO), 2018).

[5] 21NRM05 STASIS Project , “Standardisation for Safe Implant Scanning in MRI. 2022–2025,” https://www.ptb.de/stasis/.

[6] H. Bassen and T. Zaidi , “Parameters Affecting Worst‐Case Gradient‐Field Heating of Passive Conductive Implants,” Journal of Magnetic Resonance Imaging 56 (2022): 1197–1204. 35778374 10.1002/jmri.28321

[7] R. G. Sargent , “Verification and Validation of Simulation Models,” Journal of Simulation 7 (2013): 12–24, 10.1057/jos.2012.20.

[8] E. Neufeld , S. Kühn , G. Szekely , and N. Kuster , “Measurement, Simulation and Uncertainty Assessment of Implant Heating During MRI,” Physics in Medicine and Biology 54 (2009): 4151–4169, 10.1088/0031-9155/54/13/012. 19521007

[9] O. Bottauscio , A. M. Cassarà , J. W. Hand , et al., “Assessment of Computational Tools for MRI RF Dosimetry by Comparison With Measurements on a Laboratory Phantom,” Physics in Medicine and Biology 60 (2015): 5655–5680. 26147075 10.1088/0031-9155/60/14/5655

[10] H. Graf , G. Steidle , and F. Schick , “Heating of Metallic Implants and Instruments Induced by Gradient Switching in a 1.5‐Tesla Whole‐Body Unit,” Journal of Magnetic Resonance Imaging 26 (2007): 1328–1333, 10.1002/jmri.21157. 17969167

[11] K. E. Bannan , W. Handler , B. Chronik , and S. P. Salisbury , “Heating of Metallic Rods Induced by Time‐Varying Gradient Fields in MRI,” Journal of Magnetic Resonance Imaging 38 (2013): 411–416, 10.1002/jmri.23984. 23293032

[12] R. Brühl , A. Ihlenfeld , and B. Ittermann , “Gradient Heating of Bulk Metallic Implants Can Be a Safety Concern in MRI,” Magnetic Resonance in Medicine 77 (2017): 1739–1740, 10.1002/mrm.26652. 28247432

[13] A. Arduino , U. Zanovello , J. Hand , et al., “Heating of Hip Joint Implants in MRI: The Combined Effect of RF and Switched‐Gradient Fields,” Magnetic Resonance in Medicine 85, no. 6 (2021): 3447–3462. 33483979 10.1002/mrm.28666 PMC7986841

[14] M. Viceconti and L. Emili , eds., Toward Good Simulation Practice. Best Practices for the Use of Computational Modelling and Simulation in the Regulatory Process of Biomedical Products (Springer, 2024), 10.1007/978-3-031-48284-7.

[15] O. Bottauscio , M. Chiampi , J. Hand , and L. Zilberti , “A GPU Computational Code for Eddy‐Current Problems in Voxel‐Based Anatomy,” IEEE Transactions on Magnetics 51, no. 3 (2015): 5100904.

[16] A. Arduino , O. Bottauscio , R. Brühl , M. Chiampi , and L. Zilberti , “In Silico Evaluation of the Thermal Stress Induced by MRI Switched Gradient Fields in Patients With Metallic Hip Implant,” Physics in Medicine and Biology 64 (2019): 245006. 31683262 10.1088/1361-6560/ab5428

[17] A. Arduino , O. Bottauscio , M. Chiampi , and L. Zilberti , “Douglas–Gunn Method Applied to Dosimetric Assessment in Magnetic Resonance Imaging,” IEEE Transactions on Magnetics 53, no. 6 (2017): 5000204.

[18] Dassault , “CST Studio Suite,” https://www.3ds.com/products/simulia.

[19] U. Zanovello , C. Fuss , A. Arduino , and O. Bottauscio , “Efficient Prediction of MRI Gradient‐Induced Heating for Guiding Safety Testing of Conductive Implants,” Magnetic Resonance in Medicine 90 (2023): 2011–2018, 10.1002/mrm.29787. 37382200

