# Supplementary material for: Validating Numerical Simulations to Support Experimental Testing of MRI Gradient‐Induced Heating of Passive Implants
Source: Magn Reson Med. 2026 Feb 15;95(6):3584–93. doi: 10.1002/mrm.70306 (PMC13049244; doi:10.1002/mrm.70306)
Supplement: Supplementary file 1 — Data S1: mrm70306‐sup‐0001‐Supinfo.docx. [file MRM-95-3584-s001.docx]

**SUPPORTING INFORMATIONS**

**Data S1 - Characteristics of the considered implants**

The knee implant model was Genus MB (Adler Ortho® SpA, Italy). The material of the femur and tibial components is CrCoMo alloy. The liner is made of UHMWPE.

The shoulder implant model was SMR Anatomic (LimaCorporate, Italy). The material of the humeral head is CrCoMo alloy. The other implant components are made of Ti-6Al-4V.

The hip implant model was a composition from Adler Ortho® SpA, Italy of Apta-Fix (stem and ball) and Fixa Ti-Por (acetabular cup and liner). The material of the stem and the acetabular cup is Ti-6Al-4V alloy. The ball is made of CrCoMo. The liner is made of UHMWPE.

Supporting information Figure S1 shows the implant specimens, whose relevant dimensions are reported in Supporting information Table S1. The physical characteristics of the implants are summarized in Supporting information Table S2.

The gel used in the measurements performed at MR:comp is the acrylic glass phantom filled is made of 1.4 g/L NaCl (Merck, GR for analysis, Darmstadt, Germany) solved in distilled water acc. to VDE 0510 (Phönix, Bergneustadt, Germany) mixed with 10 g/L polyacrylic acid (PAA), partial sodium salt, lightly cross linked, Catalog No. 436364 (ALDRICH, St. Louis, MO, USA).

The gel used in the measurements performed at INRiM is a Zurich Med Tech TSM (TGe78c0.47@64) whose main ingredients are water, salt, and HEC (reference to ISO/ TS 10974). The physical characteristics of the two gels are also reported in Table S2.


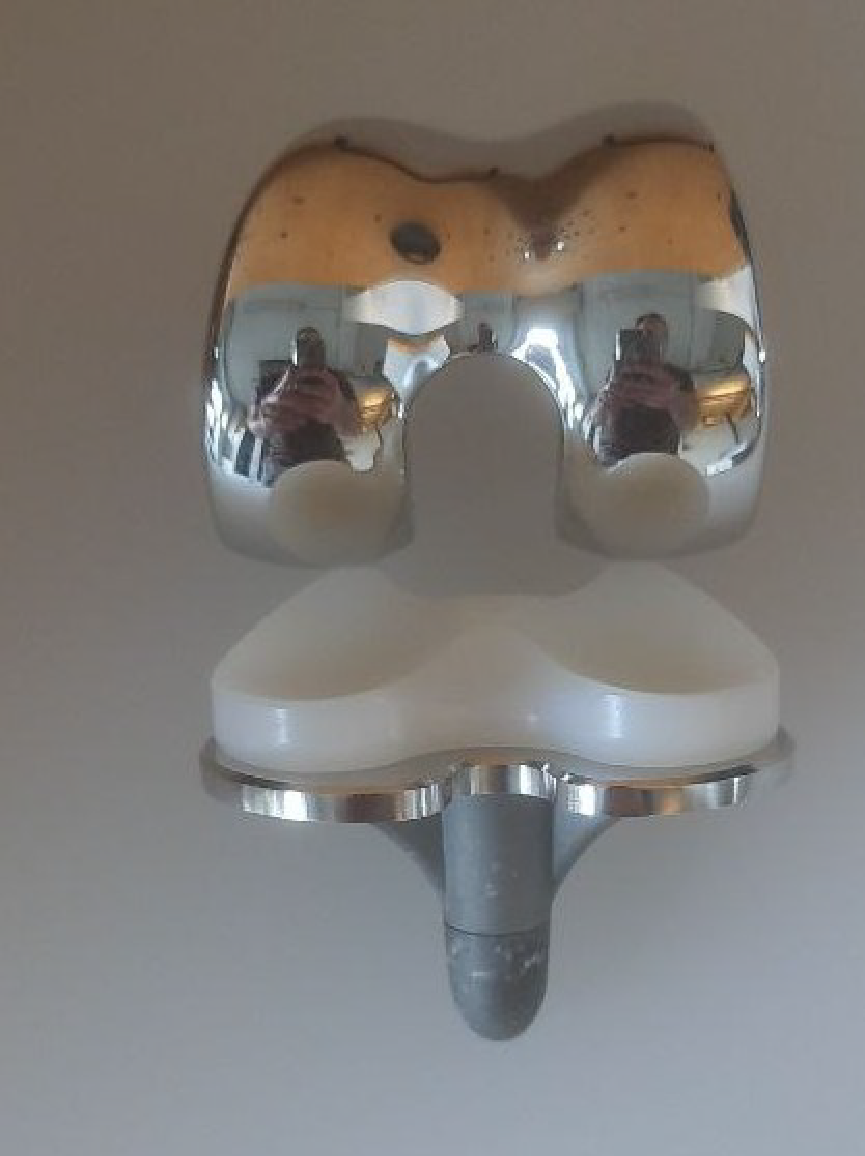

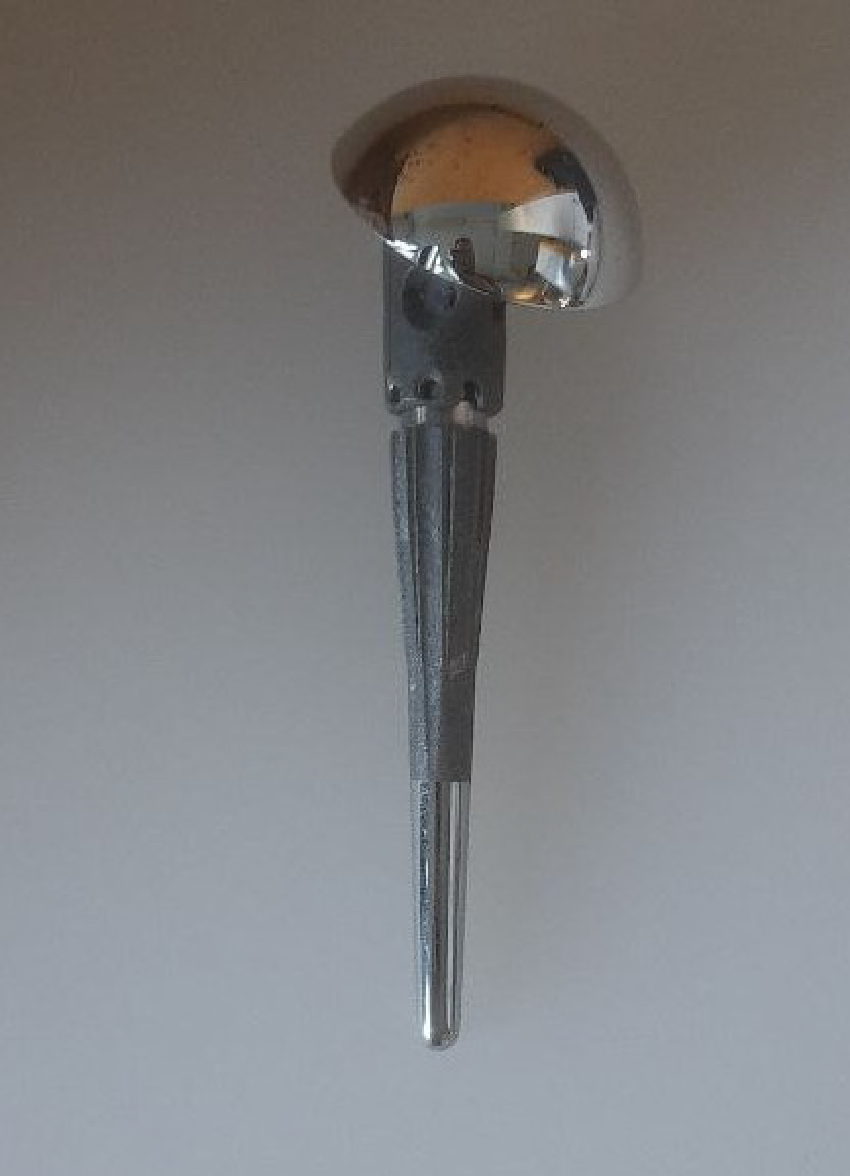

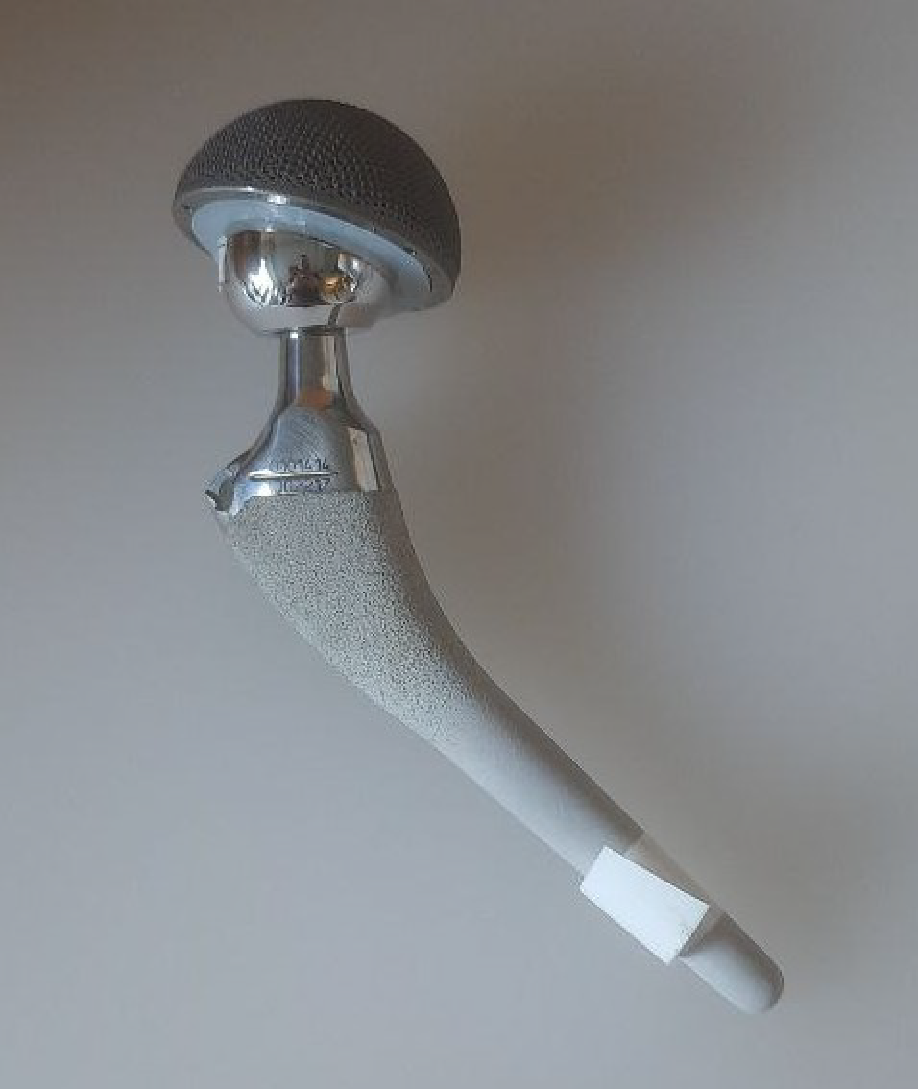


(a) (b) (c)

Figure S1 – Considered implant specimens: (a) Genus MB knee implant (Adler Ortho® SpA, Italy), (c) SMR Anatomic shoulder implant (LimaCorporate, Italy), (c) Apta-Fix and Fixa Ti-Por hip implant (Adler Ortho® SpA, Italy)

Table S1 – Relevant dimensions of the implants

| Implant | Component | Dimensions |
| --- | --- | --- |
| Hip | Stem | Volume: 34,007 mm^3^  Height: 159.4 mm  Width: 61.3 mm  Thickness: 20.7 mm |
|  | Ball | Volume: 13,726 mm^3^  Diameter: 31.7 mm |
|  | Acetabular cup | Volume: 16,190 mm^3^  External diameter: 52.1 mm |
| Knee | Femoral | Volume: 30,914 mm^3^  Height (up-down): 60.5 mm  Width (left-right): 69.6 mm  Thickness (front-rear): 51.9 mm |
|  | Tibial plate | Volume: 13,111 mm^3^  Plate height: 43.3 mm  Plate width: 73.1 mm  Thickness: 47.2 mm |
| Shoulder | Stem + Humeral body | Volume: 9,053 mm^3^  Height: 110.3 mm  Max stem diameter: 24 mm |
|  | Humeral head | Volume: 8,097 mm^3^  External diameter: 41.5 mm |

Table S2 – Physical characteristics of the materials

|  | Electrical conductivity  (S/m) | Thermal conductivity  (W/m/K) | Specific Heat capacity  (J/kg/K) | Density (kg/m^3^) |
| --- | --- | --- | --- | --- |
| CoCrMo alloy | 1.16 x 10^6^ | 14 | 450 | 8,445 |
| Ti-6Al-4V | 0.581 x 10^6^ | 7.2 | 520 | 4,420 |
| UHMWPE | 5 x 10^-4^ | 0.41 | 1,840 | 930 |
| Phantom gel | 0.43 | 0.624 | 4,200 | 1,006 |

**Data S2 - Uncertainty budget of measurements following ISO/TS 10974:2018 test method**

The measurement uncertainty associated with these sets of measurements was evaluated combining the different components. For the uncertainty related to the field source, the considered components were divided between uncertainty in the source and uncertainty in the field measurements. For the former, gradient amplifier stability and spatial uniformity were evaluated. Considering the latter, the following components associated with the magnetic field probe were accounted for: spherical isotropy, noise, sensor displacement, readout electronics, probe positioning and orientation, calibration uncertainty. The expanded uncertainty (95 % confidence interval) for dB/dt field resulted to be 3.1 %.

For the uncertainty related to temperature measurements, the considered components were: probe and data acquisition, medium parameters and test object position. The expanded uncertainty (95 % confidence interval) for temperature measurements resulted to be 14.9 %.

**Data S3 - Experimental set-up MRI gradient coil experiments**

The experimental set-up available at INRiM is based on actively shielded whole-body gradient coils (Solaris-R, Nanjing Cichen Medical Technology Co., Ltd, Nanjing, China). The gradient coil system has been designed to be used in superconducting MRI magnets of flux density up to 1.5 T. It includes three coils to generate gradient magnetic fields along three Cartesian directions.

The internal diameter of the coils is 670 mm and the length is 1,501 mm. The coils have safe operating conditions with peak current of 600 A and peak voltage of 800 V. The allowed steady-state current is 200 A. The coils are water cooled to keep the temperature of the conductors below 70 °C.

The sensitivities of the three coils are: 55.83 μT/m/A ± 2 % for the coil which generates an *X*-axis gradient, 55 μT/m/A ± 2 % for the coil which generates a *Y*-axis gradient and 59 μT/m/A ± 2 % for the coil which generates a *Z*-axis gradient. The inductances and DC resistances of the three coils are respectively 357 μH and 86 mΩ (*X*-coil), 359 μH and 90 mΩ (*Y*-coil), and 357 μH and 92 mΩ (*Z*-coil). Supporting information Figure S2 shows the coil system.


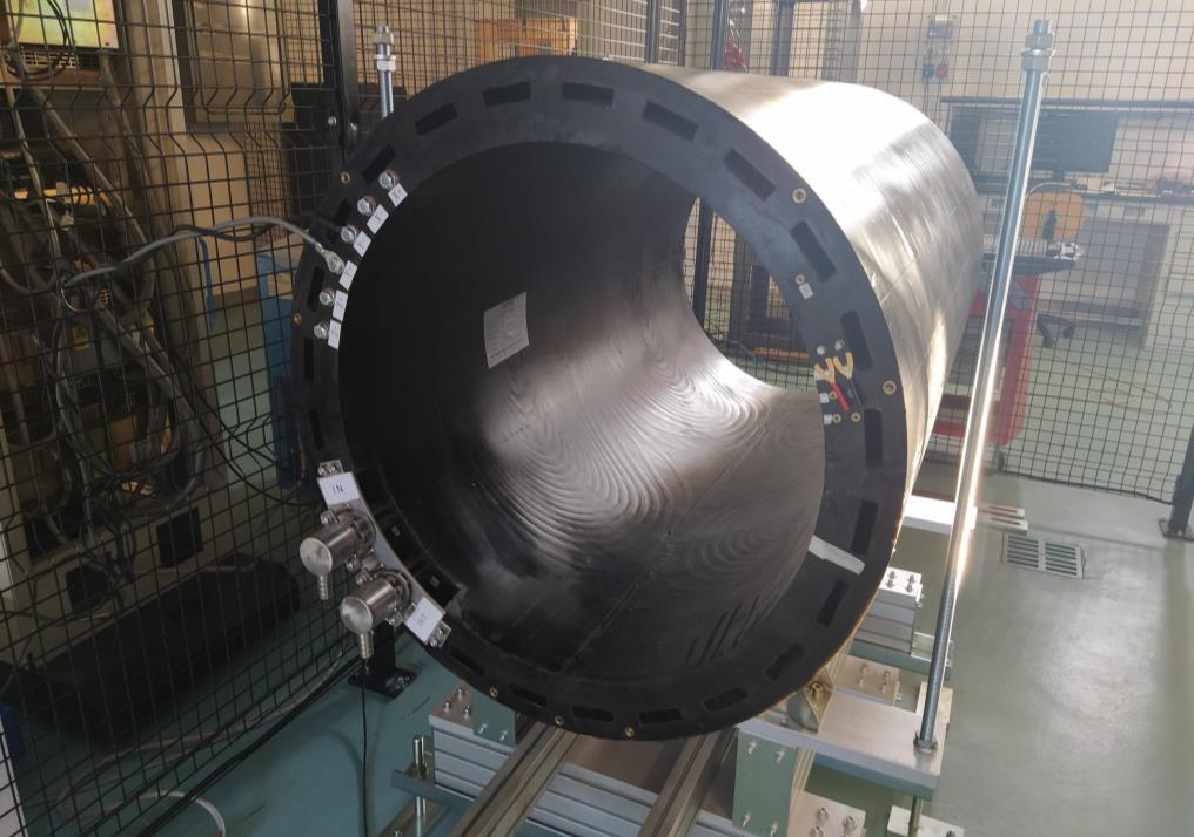


Figure S2. The three-axis gradient coil system available at INRiM laboratories

An HP-01 Magnetometer – Field, Analyzer (NARDA Safety Test Solution®, Milano, Italy) is used to preliminary map the magnetic field within the coil. The HP-01 Magnetometer is a magnetic DC and low frequency isotropic field probe-analyzer providing a solution for magnetic field analysis in a frequency range from DC to 1 kHz in a high dynamic range. It includes three Hall effect sensors for simultaneous measurements of magnetic flux density along X, Y and Z axes. In the lower range, the probe has a measurement dynamic from 10 μT to 60 mT with a resolution of 100 nT. The probe was preliminary calibrated under DC conditions by means of two Helmholtz coils with active compensation of the earth's magnetic field, which are the magnetic flux density Italian standard (KCDB Database of CMCs; Institute code: INRIM/206 and INRIM/207; Quantity: DC Magnetic Flux Density). The AC calibration was performed by means of an AC Helmholtz coil (KCDB Database of CMCs; Institute code: INRIM/208; Quantity: Magnetic Field below 50 kHz, AC Magnetic Flux Density). The probe has been calibrated at five different frequencies (60 Hz, 120 Hz, 240 Hz, 480 Hz, 960 Hz).

**Data S4 - Uncertainty budget of measurements with MRI gradient coils**

The measured value of temperature increase at each instant was estimated as the average of two repetitions of the heating experiment. Before averaging them, the measurement results of each repetition were filtered in time according to a moving average (window size of 10 time samples).

The following uncertainty sources are taken into account to determine the measurement uncertainty:

- Thermal probe positioning. Through numerical simulations, it has been quantified the variability of the temperature increase on the implant surface in a neighbourhood of the expected probe location, according to the size of the sensitive part of the probe temperature (1 mm × 2 mm × 3 mm), as declared by the manufacturer.
- Repeatability. It has been observed that the largest difference that occurred between the two repetitions was equal to 0.1 K.
- Noise. By taking the difference between the raw signal measured during each experiment and the filtered signal, it is possible to isolate the noise signal. Assuming that it is uncorrelated Gaussian noise, the uncertainty of each filtered time instant associated with the noise is evaluated as the standard deviation of the noise signal divided by the square root of the sample window size of the filter (10 samples). Finally, the uncertainty of each filtered signal is propagated through the averaging of the two repetitions.

These three contributions are combined quadratically according to the law of propagation of the uncertainty to assign the combined standard uncertainty to the measured values, from which an expanded uncertainty is deduced with a coverage factor of 2 (95 % coverage level).

**Data S5 - Additional information related to simulations of ISO/TS 10974:2018 test experiments**

The number of voxels used to describe the femoral component of the knee implant was 1,147,409 in the electromagnetic simulations and 42,464 in the thermal simulations (with a total number of voxels equal to 9,628,058 including the phantom). In the TET-based model the number of tetrahedral elements was 1,078,738 (minimum edge size of 1.44 x 10^-2^ mm) for the electromagnetic solver and 658,383 (minimum edge size of 8.35 x 10^-3^ mm) for the thermal solver (including the phantom).

The number of voxels used to describe the tibial component of the knee implant was 478,951 in the electromagnetic simulations and 19,778 in the thermal simulations (with a total number of voxels equal to 9,628,058 including the phantom). In the TET-based model the number of tetrahedral elements was 1,091,209 (minimum edge size of 3.63 x 10^-3^ mm) for the electromagnetic solver and 159,052 (minimum edge size of 5.39 x 10^-3^ mm) for the thermal solver.

The number of voxels used to describe the shoulder implant was 783,290 in the electromagnetic simulations and 29,122 in the thermal simulations (with a total number of voxels equal to 9,628,058 including the phantom). In the TET-based model the number of tetrahedral elements was 430,777 (minimum edge size of 2.43 x 10^-4^ mm) for the electromagnetic solver and 240,982 (minimum edge size of 2.43 x 10^-4^ mm) for the thermal solver.

All results are summarized in Supporting information Table S3. A good correlation between deposited power and temperature increase is evident. The ratios between results obtained with TET-based and voxel-based approaches can be mainly explained considering local variation of the induced currents due to the different discretization, rather than a global effect due to different equivalent volumes of the discretized objects.

Table S3 - Complete summary of the simulation results

|  | Femoral component | | | Tibial component | | | Shoulder | | |
| --- | --- | --- | --- | --- | --- | --- | --- | --- | --- |
|  | Volume  (m^3^) | Total power (W) | Δ*T*_max_  (K) | Volume  (m^3^) | Total power (W) | Δ*T*_max_  (K) | Volume  (m^3^) | Total power (W) | Δ*T*_max_  (K) |
| Voxel-based | 3.10 10^-5^ | 3.13 | 8.0 | 1.29 10^-5^ | 1.67 | 5.3 | 2.11 10^-5^ | 1.14 | 4.5 |
| TET-based | 3.09 10^-5^ | 2.89 | 7.1 | 1.29 10^-5^ | 1.75 | 5.7 | 2.11 10^-5^ | 1.12 | 4.1 |
| Ratio  (Voxel-based vs. TET-based | 1.002 | 1.08 | 1.12 | 1.002 | 0.96 | 0.93 | 1.005 | 1.02 | 1.12 |

**Data S6 - Positioning of the implants in the gradient coil system**


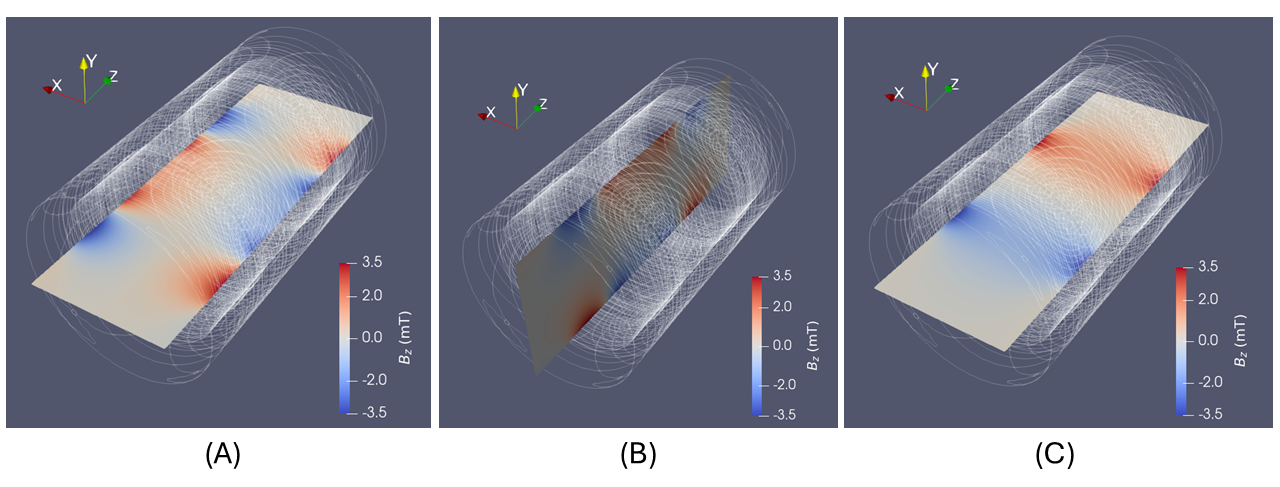


Figure S3. Maps of the z-component of the magnetic field generated by the three gradient coil axes supplied with a current equal to 100 A: (A) *X*-coil gradient, (B) *Y*-coil gradient, (C) *Z*-coil gradient.

Table S4 - Position of the implants within the gradient coil system. The origin of the cartesian system is located in the isocenter of the gradient coil system. The magnetic flux density components reported in the Table were obtained supplying the *X*-coil with a current of amplitude equal to 146.2 A and the *Z*-coil with a current of amplitude equal to 147.1 A.

| Implant | Reference point  (point labeled *P*_ref_ in Figure 1) | *X*  (mm) | *Y*  (mm) | *Z*  (mm) | *X*-coil | | | *Z*-coil | | |
| --- | --- | --- | --- | --- | --- | --- | --- | --- | --- | --- |
|  |  |  |  |  | *B*_x_ (mT) | *B*_y_ (mT) | *B*_z_ (mT) | *B*_x_ (mT) | *B*_y_ (mT) | *B*_z_ (mT) |
| Tibial component | Tibial plate hole center | 87.5 | 0.4 | -301 | 2.07 | -0.08 | 0 | 0.12 | 0.11 | 2.07 |
| Femoral component | Point close to the bifurcation of the femoral component | 87.5 | -1.1 | -301 | 2.04 | 0.06 | -0.02 | 0.10 | 0.11 | 2.04 |
| Knee | Tibial plate hole center | 87.5 | 9.0 | -311 | 2.04 | 0 | 0.15 | 0.16 | 0.11 | 2.04 |
| Shoulder | Center of the humeral head | -173.3 | -3.0 | 391 | -2.51 | -0.13 | -1.36 | 0.44 | -0.09 | -1.69 |
| Hip | Center of the ball | -91.0 | 4.26 | -283.2 | 2.15 | 0.07 | 0.12 | 0.09 | 0.18 | 2.06 |


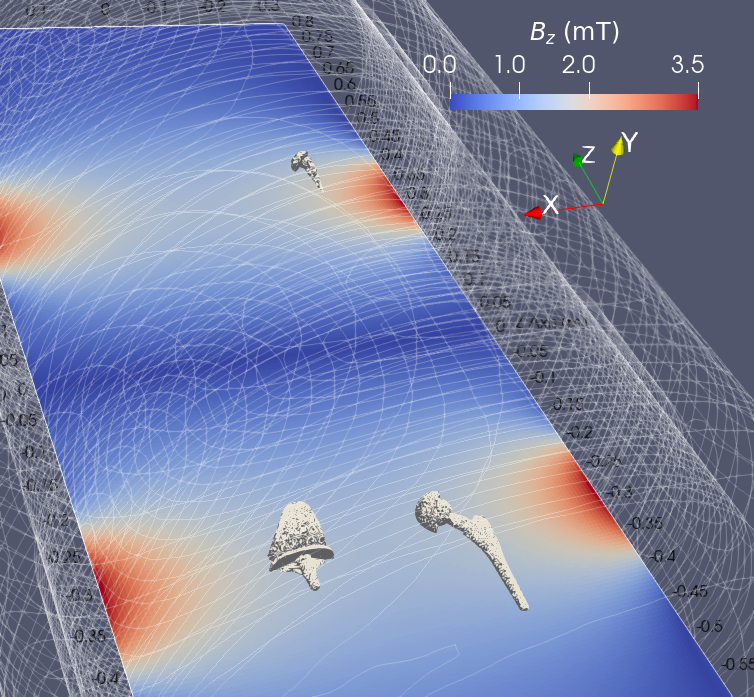


Figure S4. Position of the knee, shoulder and hip implants within the gradient coils. Implant positions have been chosen to respect the anatomical position within the body with respect to the left-rigth (*X*-axis) and front-rear (*Y*-axis) positions and trying to maximize the field amplitude with respect to the head-foot direction (*Z*-axis). The amplitude (without sign) of the *B*_z_ component of the magnetic field is shown in the colour map.


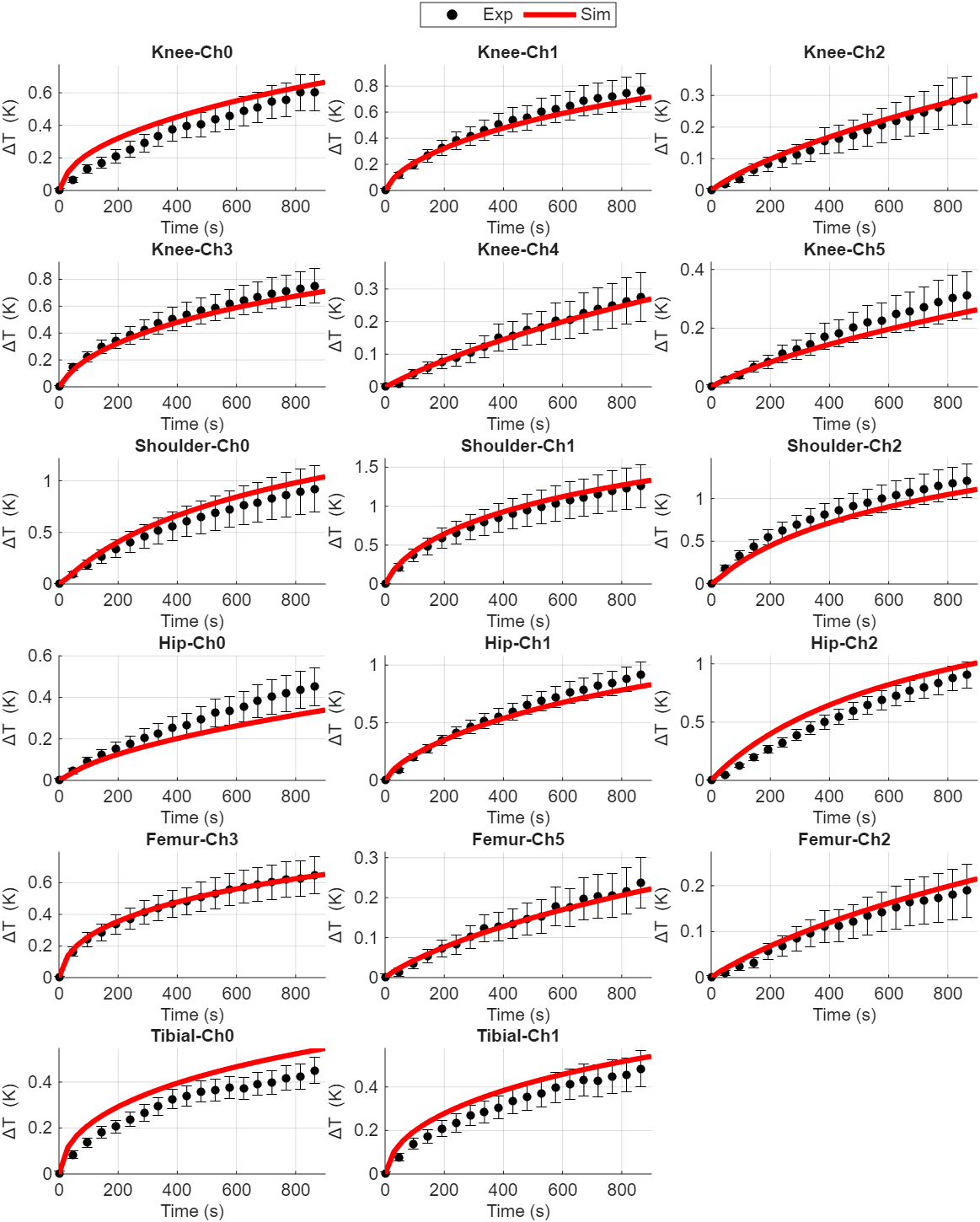


Figure S5 – Time evolution of the temperature increase for all DUTs under exposure to sinusoidal time-waveform non-uniform magnetic field. Experiments with only tibial and femoral components are also reported. For each DUT and sensor, the curves obtained by the voxel-based simulations are reported as solid lines. A selection of measured instant values, extracted from the experimental records, are plotted together with the corresponding expanded uncertainty bars (95 % level of confidence).

**Data S7 - Trapezoidal waveform used to mimic readout signal of an echo planar imaging sequence**


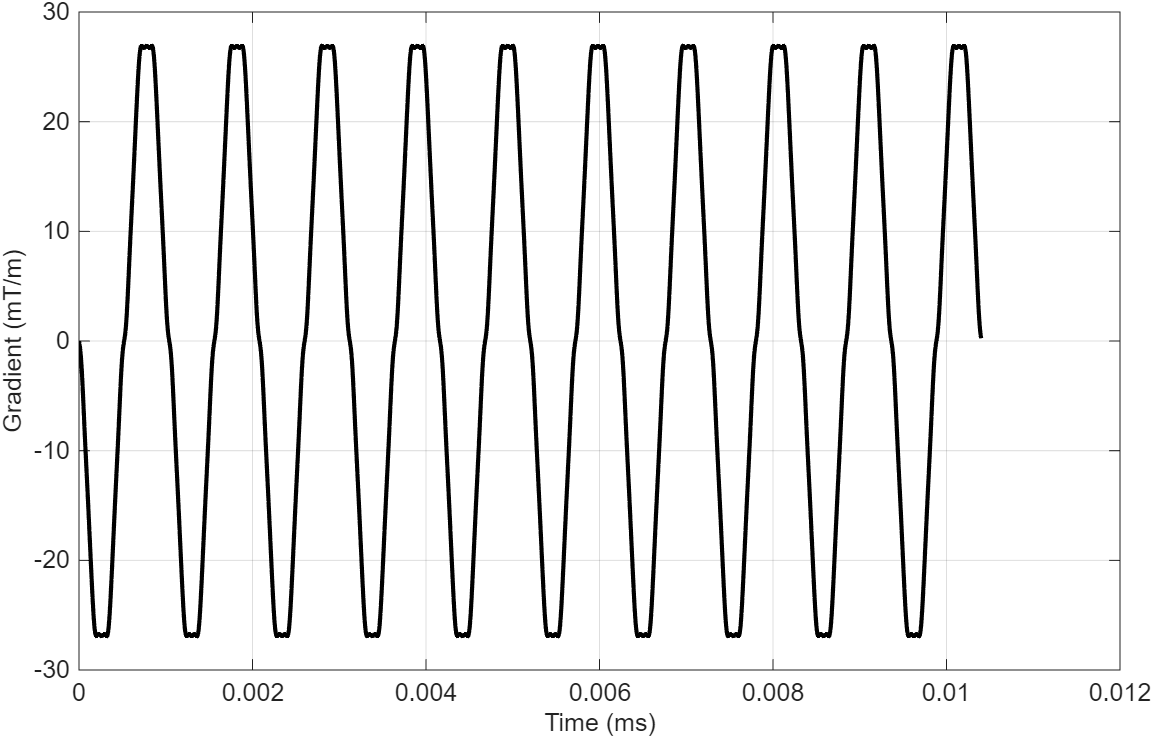


Figure S6. Trapezoidal time waveform of the magnetic gradient field used to simulate the readout signal of an echo planar imaging sequence (repetition of 10 periods). Maximum value equal to 28 mT/m. The amplitudes of the spectrum components are: 27.4 mT/m (fundamental harmonic equal to 916 Hz), 1.32 mT/m (3rd harmonic), 1.70 mT/m (5th harmonic), 0.191 mT/m (7th harmonic), 0.335 mT/m (9th harmonic), 0.348 mT/m (11th harmonic), 0 mT/m (13th harmonic), 0.110 mT/m (15th harmonic). The spectrum was truncated at the 15th harmonic as used in the simulation based on a frequency domain solver. The waveform reported in the figure is the one reconstructed after truncated Fourier transform, so that a small ripple appears in proximity of the slope changes.


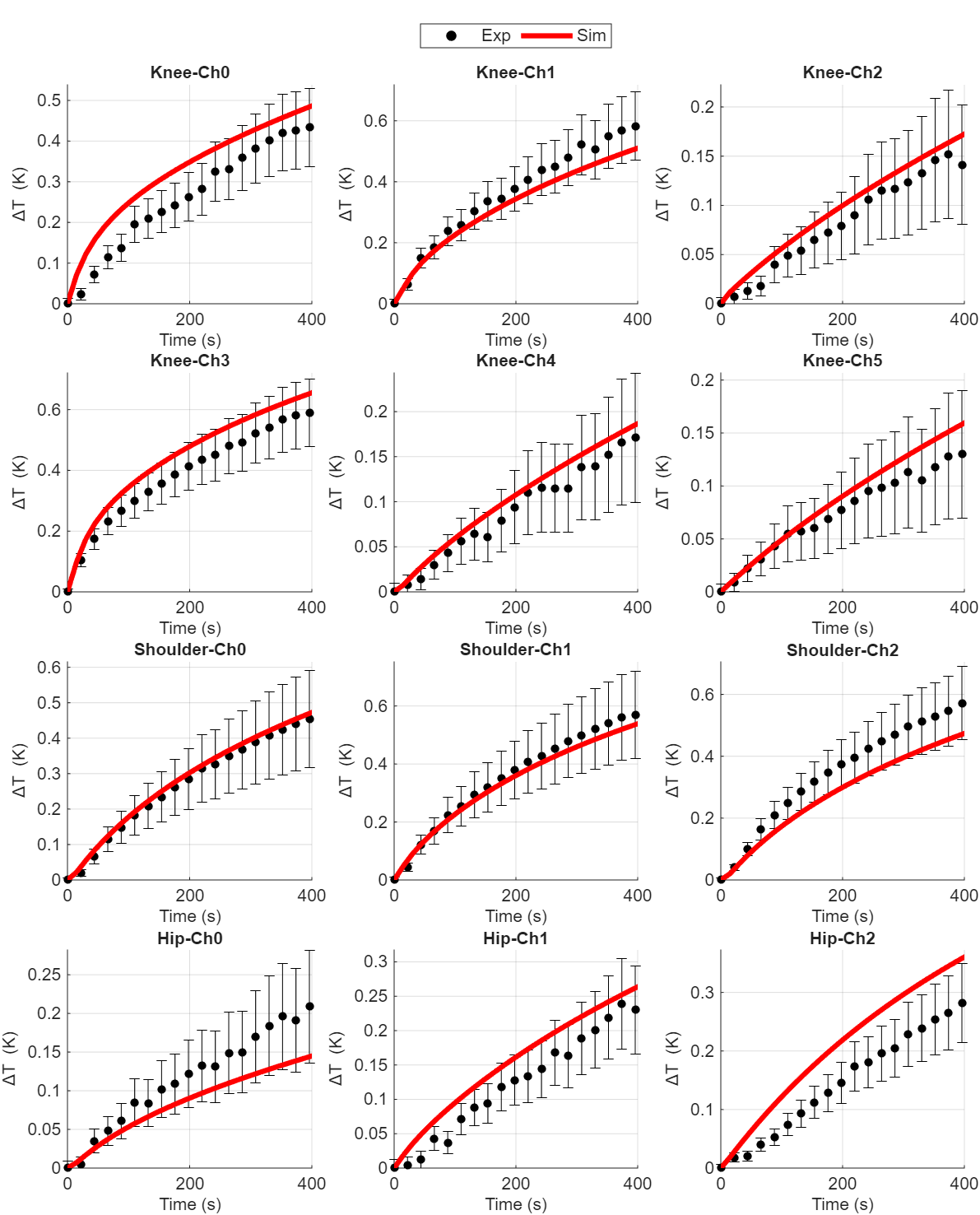


Figure S7 – Time evolution of the temperature increase for all DUTs under exposure to trapezoidal time-waveform non-uniform magnetic field. The same DUT positions of the exposure with sinusoidal waveform are reported here. For each DUT and sensor, the curves obtained by the voxel-based simulations are reported as solid lines. A selection of measured instant values, extracted from the experimental records, are plotted together with the corresponding expanded uncertainty bars (95 % level of confidence).

**Data S8 - Histograms of the global predictive inaccuracy value 𝞮**

**
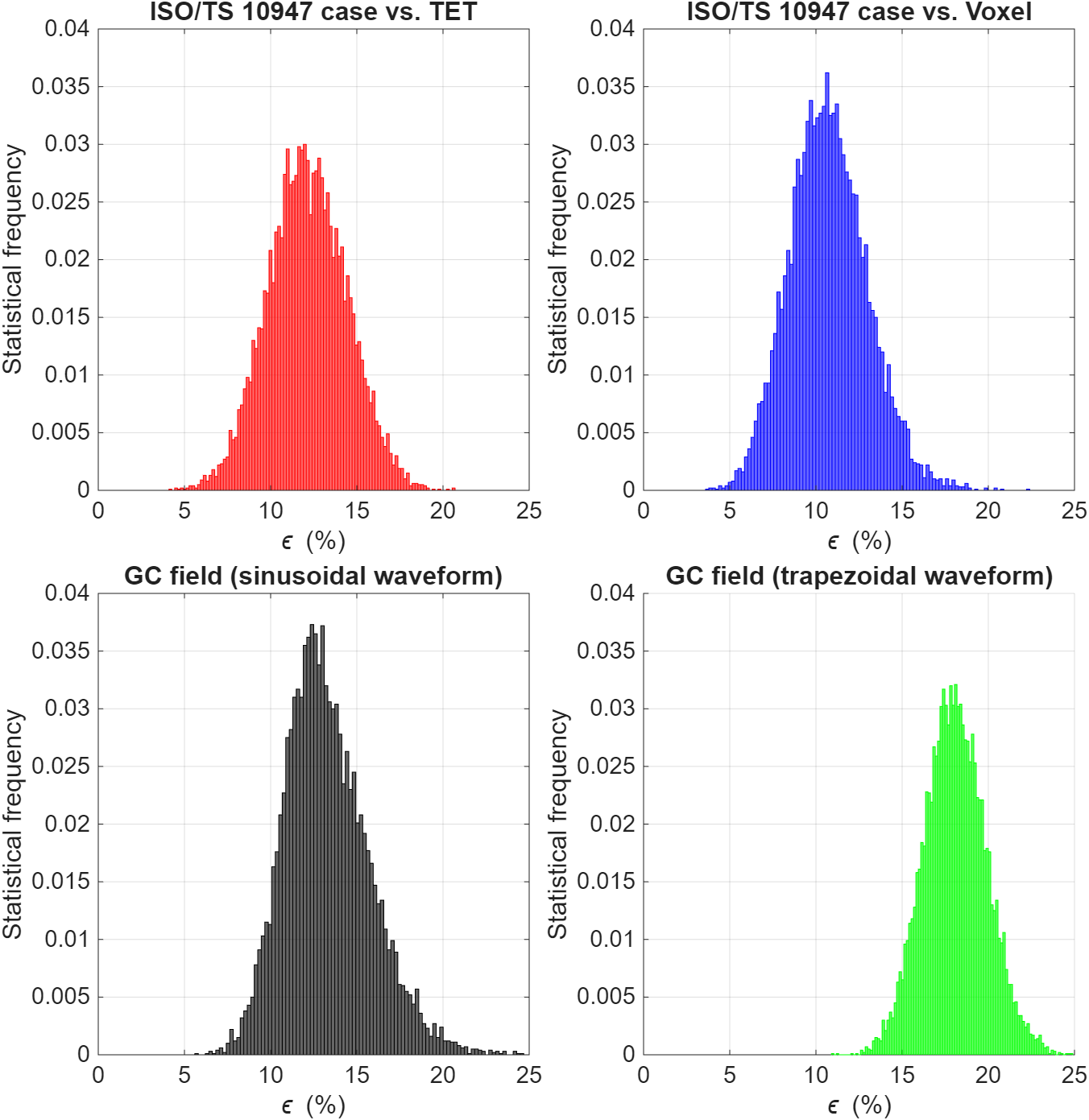
**

Figure S8. Histograms of predictive inaccuracy values 𝞮 for the ISO/TS 10974:2018 test results, the gradient coils (GC) under sinusoidal waveform results, and the gradient coils under trapezoidal waveform results.
